# A Bayesian ARMA Probability Density Estimator

**DOI:** 10.3390/e27101001

**Published:** 2025-09-26

**Authors:** Jeffrey D. Hart

**Affiliations:** Department of Statistics, Texas A&M University, College Station, TX 77843, USA; hart@stat.tamu.edu

**Keywords:** BIC, Laplace approximation, importance sampling, truncated Fourier series, identifiability, single-component Metropolis–Hastings algorithm, integrated squared error, Kullback–Leibler discrepancy, probability bands

## Abstract

A Bayesian approach for constructing ARMA probability density estimators is proposed. Such estimators are ratios of trigonometric polynomials and have a number of advantages over Fourier series estimators, including parsimony and greater efficiency under common conditions. The Bayesian approach is carried out via MCMC, the output of which can be used to obtain probability intervals for unknown parameters and the underlying density. Finite sample efficiency and methods for choosing the estimator’s smoothing parameter are considered in a simulation study, and the ideas are illustrated with data on a wine attribute.

## 1. Introduction

Density estimators based on ratios of trigonometric polynomials, or ARMA estimators [1,2], have a number of motivations, including parsimony and the fact that they mitigate the spurious modes that are a well-known deficiency of truncated Fourier series estimators. Also important is a super-efficiency result documented in [1], where it is shown that, under standard sets of conditions, a certain ARMA estimator has asymptotically smaller mean integrated squared error than that of *any* tapered Fourier series estimator. Nonetheless, ARMA density estimators have not attracted a great deal of attention. The author is unaware of any literature on the subject other than [1,2,3]. There has been some recent interest, however, in using rational functions for regression analysis [4].

One problem with the estimates considered in [1] is that they do not guarantee positivity. In this paper, we propose a Bayesian implementation of ARMA estimators that guarantees positivity but nonetheless has the attractive properties mentioned above. The Bayesian approach also leads to readily available probability intervals for unknown parameters and bands for the underlying density itself.

One might ask why it is necessary to consider ARMA density estimators in light of the preponderance of methods that exist for density estimation [5]. A main reason for doing so is the dearth of literature on such estimators in spite of the attractive properties they possess, as mentioned above. One aspect of ARMA estimators that has received scant attention is parameter estimation. In this paper, parameters are estimated via a Bayesian approach, which, of course, is essentially equivalent to maximum likelihood in large samples. ARMA estimators based on maximum likelihood promise to be more efficient than those of [1,3]. It is therefore possible that the advantages documented in [1] are actually somewhat understated.

The rest of the paper will proceed as follows: ARMA estimators and our basic approach are introduced in Section 2, while the advantages of ARMA representations over those of the truncated Fourier series variety are illustrated in Section 3. Details of our Bayesian implementation are given in Section 4, and results of a simulation study described in Section 5. A data analysis involving a wine attribute is presented in Section 6, and, finally, a few concluding remarks are given in Section 7.

## 2. ARMA Probability Density Estimators

In the following, we shall assume that a random sample $X_{1},\ldots ,X_{n}$ is available from a square integrable density *f* that has support (0,π). These data are to be used to obtain a nonparametric estimate of *f*. Let $\theta^{q}=\left(\theta_{1},\ldots ,\theta_{q}\right)$ and $\phi^{p}=\left(\phi_{1},\ldots ,\phi_{p}\right)$ be vectors of real-valued parameters. Then, an ARMA(p,q) representation of *f* has the form

(1) $$f\left(x|\theta^{q},\phi^{p}\right)\propto \frac{{\left|1-\theta_{1}e^{ix}-\cdots -\theta_{q}e^{qix}\right|}^{2}}{{\left|1-\phi_{1}e^{ix}-\cdots -\phi_{p}e^{pix}\right|}^{2}},\;\;0<x<\pi ,$$

where $i=\sqrt{-1}$. (Subsequently, *i* is used only in this way and not as an index.) The name ARMA derives from the fact that $f(\cdot |\theta^{q},\phi^{p})$ has the same form as the spectral density of an autoregressive moving average process [6]. The multiplicative constant that is needed to make $f(\cdot |\theta^{q},\phi^{p})$ integrate to 1 can be determined from $\theta^{q}$ and $\phi^{p}$ by solving a system of linear equations [6]. To ensure identifiability of the density, one may impose the condition that all zeroes of the two polynomials

(2) $$s\left(z\right)=1-\theta_{1}z-\cdots -\theta_{q}z^{q}\;\;\mathrm{and}\;\;t\left(z\right)=1-\phi_{1}z-\cdots -\phi_{p}z^{p}$$

lie outside the unit circle in the complex plane. Choosing parameters that satisfy this condition will be addressed in Section 4.

To fit ARMA representations to data, one must choose values for *p* and *q* and estimate the parameters $\phi^{p}$ and $\theta^{q}$. Our approach to parameter estimation is Bayesian. For a given *p* and *q*, we propose a prior for the unknown parameters and then explore the resulting posterior distribution by means of MCMC. For large sample sizes such an approach is essentially maximum likelihood, but with the advantage of readily available uncertainty intervals for parameters.

As for *p* and *q*, we will fix *p* at 2 and then select *q* from the data. Two means for selecting *q* are considered, one being BIC, the Bayes information criterion, and the other the principled Bayesian approach of choosing a model to maximize posterior probability. While we do not rule out the possibility of considering other choices of *p*, there are a number of advantages to using p=2. One is the obvious simplicity of having but a single smoothing parameter to select. More importantly though, the ARMA(2,q) representation by itself has qualitative and quantitative advantages over Fourier series estimators, the primary competitors of ARMA estimators. These advantages will be discussed subsequently.

It is important to appreciate the difference between the density estimators in [1] and the ones just proposed. The former estimators may be expressed as

(3) $$\hat{f}\left(x\right)=\frac{\sum_{j=0}^{q}{\hat{\beta }}_{k}\cos\left(jx\right)}{{\left|1-\alpha e^{ix}\right|}^{2}},$$

where |α|<1 and ${\hat{\beta }}_{0},\ldots ,{\hat{\beta }}_{q}$ are explicit functions of sample Fourier coefficients and α. The super-efficiency [1] of (3) is derived by regarding α as a smoothing parameter, but [1] only addressed the practical choice of α in a small simulation study. The numerator of the ARMA representation (1) may be written as $\sum_{j=0}^{q}b_{j}\left(\theta^{q}\right)\cos\left(jx\right)$, where $b_{0}\left(\theta^{q}\right),\ldots ,b_{q}\left(\theta^{q}\right)$ are explicitly determined by $\theta^{q}$. This means that (3) and (1) (for p=1) have the same basic form, but there is a fundamental difference between the two. Unlike (1), (3) can take on negative values. One of the main points of this paper is to show that the positivity of our ARMA estimators does not prevent them from achieving the desirable properties of (3).

Estimator (3) and, as we will show, our ARMA estimators may be motivated in several ways, the principal of which are the following:

- **Super-efficiency:** Suppose that $f^{\prime \prime }$ is square integrable and that one and only one of $f^{\prime }\left(0+\right)$ and $f^{\prime }\left(\pi -\right)$ is 0. Then, refs. [1,7,8] the asymptotic mean integrated squared error (MISE) of (3) with an optimum α is smaller than that of *any* Fourier series estimator, regardless of the taper used. For two additional settings, a similar super-efficiency result [1] can be attained by using a version of (3) with denominator ${\left|1-\alpha_{1}e^{ix}-\alpha_{2}e^{2ix}\right|}^{2}$. One setting is that just discussed but with both $f^{\prime }\left(0+\right)$ and $f^{\prime }\left(\pi -\right)$ nonzero, and the other is when $f^{\prime }$ has a singularity in the interval (0,π), rather than at the boundary. An example of the latter case is a density, such as the Laplace, that has a cusp.
- **Parsimony:** The efficiency gains discussed immediately above result even though (3) uses [1,2] considerably fewer parameters than truncated cosine series estimators. For example, in the case where $f^{\prime }\left(0+\right)\neq 0$, $f^{\prime }\left(\pi -\right)=0$ and $f^{\prime \prime }$ is square integrable, the optimum version of (3) uses only about 39% as many Fourier coefficients as does the best truncated series estimator. Parsimony can lead to better resolution of peaks when the sample size is not sufficiently large.
- **Removal of spurious modes:** Truncated Fourier series estimators often contain spurious modes, mainly when the underlying density has a sharp peak. Our ARMA(2,q) estimator mitigates this problem since the autoregressive component can effectively deal with the sharp peak, leaving the moving average component to deal with more subtle features.

## 3. Illustrating Advantages of ARMA Representations

Here, we will compare the performance of ARMA and Fourier series representations with respect to integrated squared error and qualitative features. The comparison allows us to illustrate notions discussed in the previous section. The (cosine) Fourier series of a square integrable density *f* with support (0,π) is

$$f\left(x\right)=\pi^{-1}\left[1+2\sum_{j=1}^{\infty }a_{j}\cos\left(jx\right)\right],\;\;0<x<\pi ,$$

where the Fourier coefficients $a_{1},a_{2},\ldots$ are

$$a_{j}=\int_{0}^{\pi }f\left(x\right)\cos\left(jx\right)\;dx,\;\;j=1,2\ldots .$$

A truncated series representation of *f* is

$$f_{m}\left(x\right)=\pi^{-1}\left[1+2\sum_{j=1}^{m}a_{j}\cos\left(jx\right)\right],\;\;0<x<\pi ,$$

and has the property that it minimizes the integrated squared error (ISE) among all linear combinations of cos(jx), j=0,1,…,m. This attractive property is tempered by the observation that $f_{m}$ need not be nonnegative, a decidedly unattractive aspect of a density representation.

A moving average (MA) representation for *f* has the form

$$\begin{array}{cc} f(x|q,\theta ) & =C_{\theta }{\left|1-\theta_{1}e^{ix}-\cdots -\theta_{q}e^{iqx}\right|}^{2}\\ \; & =\pi^{-1}\left[1+2\sum_{j=1}^{q}a_{j}\left(\theta \right)\cos\left(jx\right)\right], \end{array}$$

where each $a_{j}\left(\theta \right)$ is a function of $\theta_{1},\ldots ,\theta_{q}$. Inasmuch as f(·|q,θ) is a linear combination of cosines, it must have an integrated squared error at least as large as that of $f_{q}$. On the other hand, f(·|q,θ) is guaranteed to be a density. We will compare plots and ISE of $f_{m}$ and f(·|q,θ) for various choices of *m* and *q* in a case where *f* is rather peaked, a situation where the spurious modes of $f_{m}$ are prominent.

We consider a wrapped normal distribution for *f*. Such densities have been investigated in the context of circular data [9]. Let ϕ be a standard normal density and suppose that *X* is normally distributed with mean μ and standard deviation σ. Then, a wrapped version, $X_{W}$, of *X* is defined by

$$X_{W}=X\;\mathrm{mod}\;\pi ,$$

which has density

$$f\left(x\right)=\sum_{k=-\infty }^{\infty }\sigma^{-1}\phi \left(\frac{x+k\pi -\mu }{\sigma }\right),\;\;0<x<\pi .$$

**Figure 1:**
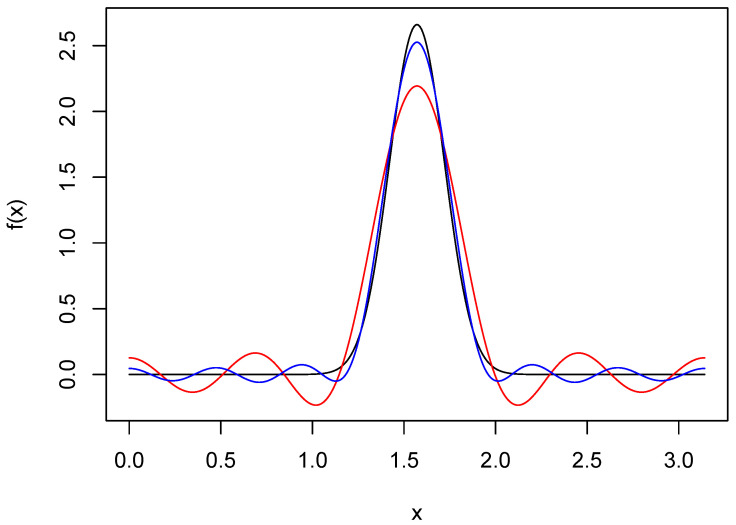
Fourier series approximations of wrapped normal density. The black curve is the wrapped normal density *f* with μ=π/2 and σ=0.15. The red and blue curves are Fourier series approximations of *f* based on truncation points 8 and 12, respectively.

A plot of this density when μ=π/2 and σ=0.15 is seen in Figure 1. Also seen there are Fourier series approximations $f_{8}$ and $f_{12}$. The latter of these does a good job of resolving the peak, but both $f_{8}$ and $f_{12}$ have spurious modes in the tails. These features are due, of course, to the fact that the cosine series may be expressed as a convolution of *f* and the Dirichlet kernel, which has oscillating tails.

**Figure 2:**
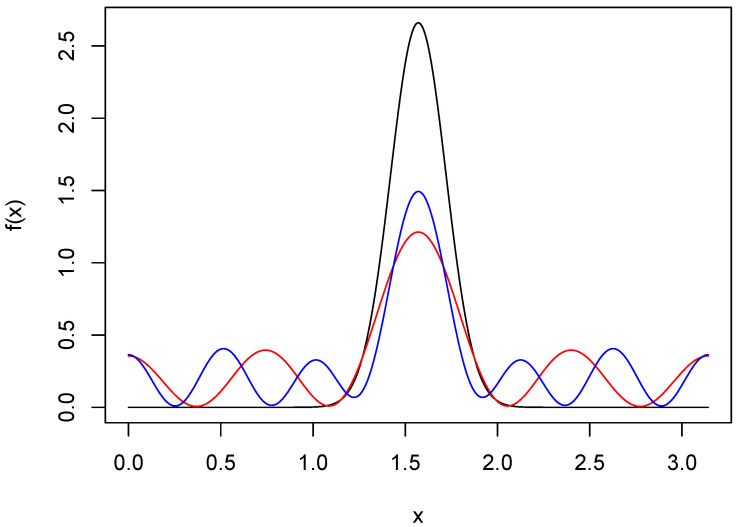
Moving average approximations of wrapped normal density. The black curve is the wrapped normal density *f* with μ=π/2 and σ=0.15. The red and blue curves are MA approximations of *f* based on 8 and 12 terms, respectively.

Let $f(\;\cdot \;|q,\theta^{\min})$ be the MA(q) representation for *f* that minimizes ISE. To see the price paid for guaranteed positivity, we show in Figure 2 $f(\;\cdot \;|8,\theta^{\min})$ and $f(\;\cdot \;|12,\theta^{\min})$ in the case of the wrapped normal density. The resolution of the peaks has been greatly compromised, and there are also oscillations in the tails.

**Figure 3:**
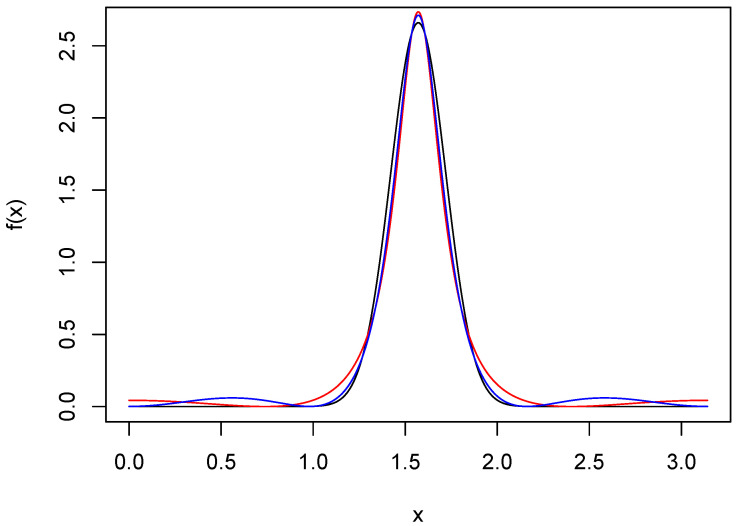
ARMA approximations of wrapped normal density. The black curve is the wrapped normal density *f* with μ=π/2 and σ=0.15. The red and blue curves are ARMA(2,4) and ARMA(2,6) approximations, respectively.

ARMA approximations of orders (2,4) and (2,6) are shown in Figure 3. The resolution of the peak is excellent for both, and the spurious modes in the tails have been greatly mitigated. The ISEs for all the approximants shown in the three figures were computed and are given in Table 1. Most noteworthy is the fact that the ISE of the ARMA(2,6) approximant is ten times smaller than that of $f_{8}$, even though both are based on the same number of parameters. This, of course, is not a contradiction, since the ARMA(2,6) approximant does not have a truncated series, but rather one for which the Fourier coefficients decay to 0 geometrically. The approximant $f_{12}$ has an ISE comparable to that of the ARMA(2,6), but requires four more parameters, thus illustrating the parsimony possible with ARMA approximants.

## 4. Implementation

In order for the parameterization in (1) to be identifiable, it is necessary that the polynomials (2) have zeroes that lie outside the unit circle. Determining the corresponding parameter space is awkward at best, which makes use of this parameterization problematic in a Bayesian context. Fortunately, an alternative parameterization that neatly takes care of this problem was discovered [10]. Let $S_{q}=\left\{\left(\psi_{1},\ldots ,\psi_{q}\right):-1<\psi_{i}<1,i=1,\ldots ,q\right\}$. Then, there exists [10] a 1–1 mapping from $S_{q}$ to the set of all $\theta^{q}$ that satisfy the identifiability constraint. If $\theta_{1},\ldots ,\theta_{q}$ were the parameters of a stationary autoregressive process (in the time series context), the corresponding $\psi_{i}$s would be the partial autocorrelations of the process [6]. The parameterization in terms of partial autocorrelations has been used [11] in a Bayesian modeling of stationary autoregressive time series.

Letting $\psi^{q}\in S_{q}$ and $\rho \in S_{2}$, the mappings into the identifiable parameter spaces of $\theta^{q}$ and ϕ will be denoted $g\left(\psi^{q}\right)=\left(\theta_{1},\ldots ,\theta_{q}\right)$ and $h\left(\rho \right)=\left(\phi_{1},\phi_{2}\right)$. We assume that the q+2 components of $\psi^{q}$ and ρ are a priori independent and identically distributed with common uniform distribution on (−1,1). The posterior distribution $\pi (\psi^{q},\rho |x_{n})$ is therefore such that

$$\pi \left(\psi^{q},\rho |x_{n}\right)\propto L\left(\psi^{q},\rho \right)={\left[C\left(\psi^{q},\rho \right)\right]}^{n}\prod_{j=1}^{n}\frac{{\left|1-\theta_{1}e^{ix_{j}}-\cdots -\theta_{q}e^{qix_{j}}\right|}^{2}}{{\left|1-\phi_{1}e^{ix_{j}}-\phi_{2}e^{2ix_{j}}\right|}^{2}},$$

where $x_{n}$ is the vector $x_{1},\ldots ,x_{n}$ of data and $C(\psi^{q},\rho )$ is the multiplicative constant needed to make $f(\;\cdot \;|g\left(\psi^{q}\right),h\left(\rho \right))$ a density.

The posterior distribution will be approximated using a single-component Metropolis–Hastings version of MCMC. Proposal distributions for the components of $\psi^{q}$ and ρ are based on rescaled and shifted beta densities. Let −1<μ<1 and 0<s<1, and take

(4) $$a=\frac{\left(1+\mu \right)}{2}\frac{\left(1-s\right)}{s}\;\;\mathrm{and}\;\;b=\frac{\left(1-\mu \right)}{2}\frac{\left(1-s\right)}{s}.$$

If *U* is a random variable with beta distribution having parameters *a* and *b* as defined in (4), then the random variable 2U−1 has support (−1,1), mean μ, and variance s(1+μ)(1−μ). We shall use r(·|μ,s) to denote the density of 2U−1.

At each iteration of the Markov chain, parameters are updated one at a time in the order $\psi_{1},\ldots ,\psi_{q},\rho_{1},\rho_{2}$. At the *j*th stage of an iteration, a proposal distribution of the form $r(\;\cdot \;|\mu ,s_{j})$ is used, j=1,…,q+2. At iteration *t*, suppose that the chain is in state $\left(\psi_{1t},\ldots ,\psi_{qt},\rho_{1t},\rho_{2t}\right)$. The first step of iteration t+1 generates a candidate $\psi_{1}^{c}$ for $\psi_{1}$ from a proposal distribution with density $r(\;\cdot \;|\psi_{1t},s_{1})$. The candidate is accepted with probability equal to the smaller of 1 and

$$\frac{\pi (\psi_{1}^{c},\psi_{2t}\ldots ,\psi_{qt},\rho_{1t},\rho_{2t}|x_{n})}{\pi (\psi_{1t},\ldots ,\psi_{qt},\rho_{1t},\rho_{2t}|x_{n})}\cdot \frac{r(\psi_{1t}|\psi_{1}^{c},s_{1})}{r(\psi_{1}^{c}|\psi_{1t},s_{1})}.$$

At subsequent stages of iteration t+1, parameters previous to the one being updated are at their updated values, while parameters after the one being updated are at their iteration *t* values. For example, suppose that $\rho_{1}$ is being updated. Then, a candidate $\rho_{1}^{c}$ is generated from $r(\;\cdot \;|\rho_{jt},s_{q+1})$ and is accepted with probability equal to the smaller of 1 and

$$\begin{array}{c} \frac{\pi (\psi_{1(t+1)},\ldots ,\psi_{q(t+1)},\rho_{1}^{c},\rho_{2t}|x_{n})}{\pi (\psi_{1(t+1)},\ldots ,\psi_{q(t+1)},\rho_{1t},\rho_{2t}|x_{n})}\cdot \frac{r(\rho_{1t}|\rho_{1}^{c},s_{q+1})}{r(\rho_{1}^{c}|\rho_{1t},s_{q+1})}. \end{array}$$

In principle, the scale parameters $s_{1},\ldots ,s_{q+2}$ could be chosen differently to produce the best mixing, but the author has had success using a single, well-chosen value of *s*.

Nonparametric density estimation always requires the choice of at least one smoothing parameter, whose role is played by *q* in our methodology. One method of choosing *q* is to use the Bayes Information Criterion, or BIC. Letting $\left({\hat{\psi }}^{q},\hat{\rho }\right)$ be the maximum likelihood estimate of $\left(\psi^{q},\rho \right)$, the BIC is defined by

$$BIC\left(q\right)=q\log\left(n\right)-2\log\left(L\left({\hat{\psi }}^{q},\hat{\rho }\right)\right),\;\;q=0,1,\ldots ,q_{max}.$$

The BIC choice of *q* is the one that minimizes BIC. For each *q*, an excellent approximation of the maximum likelihood estimate is obtained by using the parameters that maximize the likelihood over a large number of draws from the posterior distribution.

The principled Bayesian approach to choosing *q* would be to assign prior probabilities to each model and then select the *q* that maximizes the posterior probability. In a general context, suppose the models being considered are $M_{1},\ldots ,M_{k}$. The marginal likelihood of model $M_{j}$ with observed data x, parameter $\eta_{j}$, prior $\pi_{j}$ and observed likelihood $L_{j}$ is

(5) $$m_{j}\left(x\right)=\int L_{j}\left(\eta_{j}\right)\pi_{j}\left(\eta_{j}\right)\;d\eta_{j},$$

and the posterior probability of model $M_{j}$ is

(6) $$p\left(M_{j}|x\right)=p_{j}m_{j}\left(x\right){\left(\sum_{\ell =1}^{k}p_{\ell }m_{\ell }\left(x\right)\right)}^{-1},$$

where $p_{1},\ldots ,p_{k}$ are the prior model probabilities.

When the number of parameters is large, computation of the marginal likelihoods can be challenging. However, good approximations of these quantities are often possible using the method of Laplace [12]. A Laplace approximation of $m_{j}\left(x\right)$ is

$${\tilde{m}}_{j}\left(x\right)={\left(2\pi \right)}^{q_{j}/2}L_{j}\left({\hat{\eta }}_{j}\right)\pi_{j}\left({\hat{\eta }}_{j}\right)\det{\left(V_{j}\right)}^{1/2},$$

where $q_{j}$ is the dimensionality of $\eta_{j}$, ${\hat{\eta }}_{j}$ is the maximum likelihood estimate (MLE) of $\eta_{j}$, and $V_{j}$ is the covariance matrix of ${\hat{\eta }}_{j}$. The more crude approximation of $m_{j}\left(x\right)$ provided by BIC is $L_{j}\left({\hat{\eta }}_{j}\right)n^{-q_{j}/2}$. Our subsequent numerical results will compare BIC and a posterior probability approach that uses Laplace approximations of marginal likelihoods. We will refer to the latter method as Laplace.

## 5. Simulations

**Figure 4:**
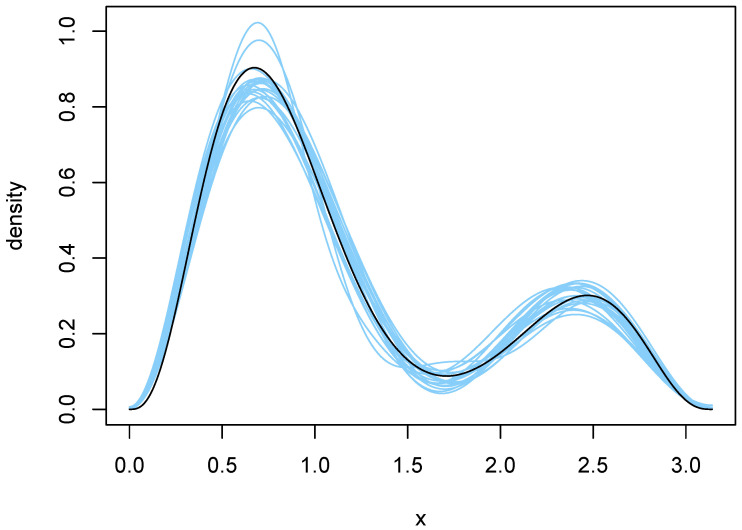
Density estimates resulting from use of BIC. The blue curves are the twenty density estimates chosen by BIC, and the black curve is the true density *f*.

**Figure 5:**
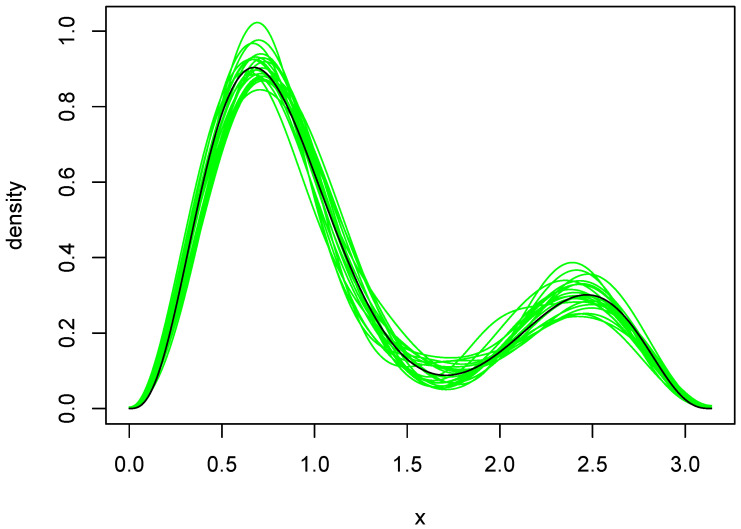
Density estimates resulting from use of the Laplace method. The green curves are the twenty density estimates chosen by the Laplace method, and the black curve is the true density *f*.

An initial small simulation was conducted to gain some insight about our MCMC procedure and the relative performance of schemes for choosing the moving average order. Because several large samples are drawn from posteriors for each dataset, only 20 replications were considered. Letting b(·|a,b) be a beta density with parameters *a* and *b*, and defining density *g* by

g(u)=0.75b(u|4,12)+0.25b(u|12,4),

the subject of our simulation is f(x)=g(x/π)/π, 0<x<π, which is the black curve in Figure 4 and Figure 5. Twenty independent random samples of n=500 each were drawn from *f*.

**Figure 6:**
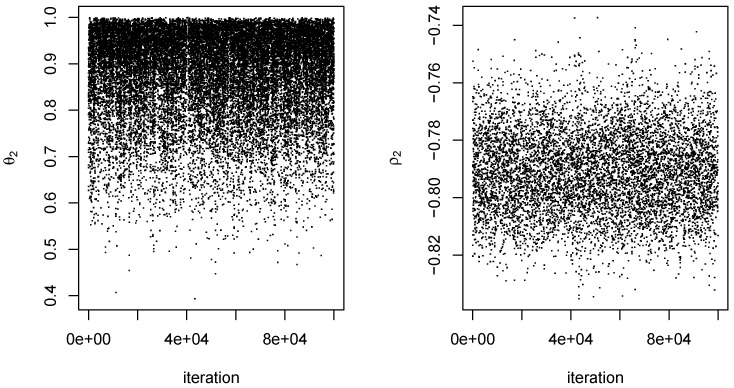
MCMC output for q=2 from one dataset. Outputs on the left and right are for the parameters $\theta_{2}$ and $\rho_{2}$, respectively. Iteration is an index for the 100,000 steps of the Markov chain.

**Figure 7:**
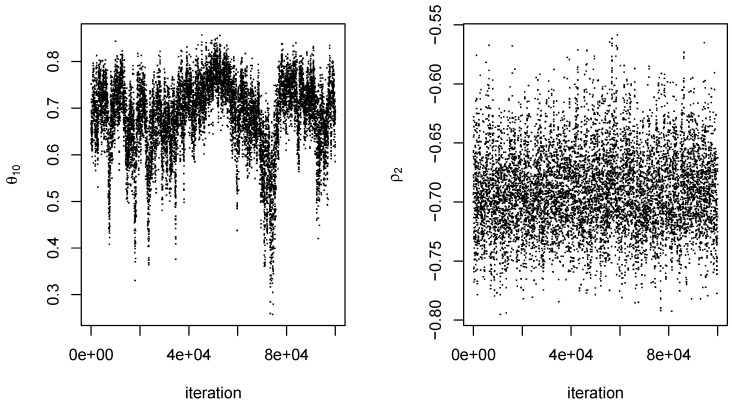
MCMC output for q=10 from the same dataset as in Figure 6. Outputs on the left and right are for parameters $\theta_{10}$ and $\rho_{2}$, respectively.

For each dataset, an MCMC procedure as described in Section 4 was conducted for q=0,1,…,10. The value of the scale parameter *s* was 0.10 for all proposal distributions. For each *q*, an initial set of 10,000 iterations was conducted using starting values of 0 for all components of $\left(\psi^{q},\rho \right)$. Because of the arbitrariness of the starting values, there was a fairly long burn-in period for at least some values of *q*. We therefore obtained parameter estimates from the initial 10,000 iterations and used them as starting values in a second set of 100,000 iterations, which was the output ultimately used to obtain our results. Mixing of $\phi_{1}$ and $\phi_{2}$ tended to be somewhat better than that of the components of $\theta^{q}$. In addition, not surprisingly, mixing of the output was better at smaller *q* than at larger ones. In any event, 100,000 iterations appeared to be more than adequate, even when q=10. Typical examples of the output obtained are given in Figure 6 and Figure 7.

For a given dataset and each *q*, the value of BIC(q) was calculated using the maximum of all likelihoods over the 100,000 iterations for that *q*. In addition, posterior probabilities were approximated using the Laplace method, as discussed in Section 4. The requisite covariance matrices in the latter method were estimated by sample covariance matrices of MCMC output.

The orders selected by BIC and the Laplace method are shown in Table 2. BIC was remarkably stable, choosing q=4 in 18 of the 20 cases. The Laplace method was somewhat more variable and yielded an average *q* of 7.4. Plots of the 20 density estimates chosen by each method are shown in Figure 4 and Figure 5. The parameters of each density were obtained by averaging over all MCMC outputs at the selected value of *q*. Both methods capture the two main features of the true density, but the estimates chosen by the Laplace method are somewhat more variable, reflecting the larger variability of the Laplace method in selecting *q*.

To further compare the two sets of density estimates, we calculated Kullback–Leibler (KL) divergences of the estimates from the true density. These quantities were approximated by numerical integration, using the function integrate in [13]. The average divergences were 0.00776 and 0.00736 for BIC and Laplace, respectively. Furthermore, the KL divergence of the Laplace estimate was smaller than that of the BIC estimate in 14 of the 20 cases. So, in spite of being somewhat more variable, the Laplace estimates tended to be a bit closer to the truth. Close examination of Figure 4 and Figure 5 reveals why this is so. The BIC estimates are more biased near the larger peak, tending to undershoot it slightly.

A second set of simulations was conducted to investigate whether or not the proposed ARMA estimators can achieve the super-efficiency of (3). Two main factors affect the efficiency of an ARMA estimator: its order and the method of parameter estimation. In this simulation, our focus was on the effect of the latter. The subject of our second simulation was the following wrapped exponential density:

(7) $$f\left(x\right)=2e^{-2x}\left(1+e^{-4(\pi -x)}\right){\left(1-e^{-4\pi }\right)}^{-1},\;\;0<x<\pi .$$

This density has a square integrable second derivative and is such that $f^{\prime }\left(0+\right)=-4$, f(π−)=0, and therefore satisfies the conditions guaranteeing super-efficiency of (3). Data may be generated from (7) by first generating data from an exponential density with rate parameter 2 and then using the wrapped construction detailed in [1].

We compared the observed ISE of ARMA(1,q) and ARMA(2,q) estimators with that of a truncated series estimator. Four sample sizes, n=250,500,1000 and 2000, were considered, with 1000 replications performed at each sample size. Asymptotically optimal orders (Theorem 1, [1]) for the truncated series estimator and (3) were $m_{n}$ and ${\tilde{m}}_{n}$, respectively. Evaluating these two orders at the wrapped exponential density (7) yielded

$$m_{n}=2.38n^{1/4}\;\mathrm{and}\;{\tilde{m}}_{n}=0.92n^{1/4}.$$

Interpreting the last two expressions as being rounded to the nearest integer, both ARMA estimators in our simulation used a moving average term of order ${\tilde{m}}_{n}$. Parameters of the two models were estimated by maximum likelihood, which was accomplished via the function optim in [13]. The series estimator used the truncation point $m_{n}$.

The ISE results are summarized in Table 3. Both the mean and median ISE of the ARMA estimators were uniformly smaller than the corresponding values for the simple series estimator. Also provided in Table 3 are asymptotic approximations [1] to the MISE of the very best tapered series estimator. At n=2000, the ratio of mean ISE for ARMA$\left(1,{\tilde{m}}_{n}\right)$ to optimum MISE was 0.776, which compares favorably with the asymptotic relative efficiency of 0.629 reported in [1]. Inasmuch as the ARMA(1,q) estimator is a special case of the ARMA(2,q), one would expect the latter estimator to also have the super-efficiency property. Indeed, this appears to be the case. A small price in terms of MISE is paid by using ARMA$\left(2,{\tilde{m}}_{n}\right)$ when ARMA$\left(1,{\tilde{m}}_{n}\right)$ is ideal, but this should be weighed against the advantage of using an estimator that can also deal effectively with a peak in the interval (0,π).

Another way to show that maximum likelihood estimation is behaving well is to examine the estimates of AR parameters. For the density (7), the asymptotically optimum choice [1] for α in estimator (3) is $1-1.39/{\tilde{m}}_{n}$. Since ${\tilde{m}}_{n}$ increases without bound as *n* increases, the optimum α tends to 1, albeit quite slowly. For the four sample sizes in our simulation, the values of $1-1.39/{\tilde{m}}_{n}$ were 0.62, 0.68, 0.73, and 0.77. For the ARMA$\left(1,{\tilde{m}}_{n}\right)$ estimator, the averages of the MLE ${\hat{\phi }}_{1}$ were 0.69, 0.71, 0.75, and 0.79. Hence, maximum likelihood seems to be doing a good job of tracking the parameters that produce super-efficiency. A similar conclusion is drawn about the ARMA$\left(2,{\tilde{m}}_{n}\right)$ estimator upon examining the roots of the polynomial $1-\phi_{1}z-\phi_{2}z^{2}$ obtained by averaging over all estimates $\left({\hat{\phi }}_{1},{\hat{\phi }}_{2}\right)$. In all four cases, this polynomial had the form $\left(1-a_{1}z\right)\left(1-a_{2}z\right)$, where $a_{1}$ and $a_{2}$ are real with different signs. For an increasing sample size, the four pairs of $\left(a_{1},a_{2}\right)$ were (0.68,−0.19), (0.70,−0.04), (0.74,−0.18), and (0.78,−0.21). As in the first-order AR case, this indicates that MLEs are doing a good job of tracking the super-efficient AR parameters.

## 6. Analysis of Wine Attribute Data

**Figure 8:**
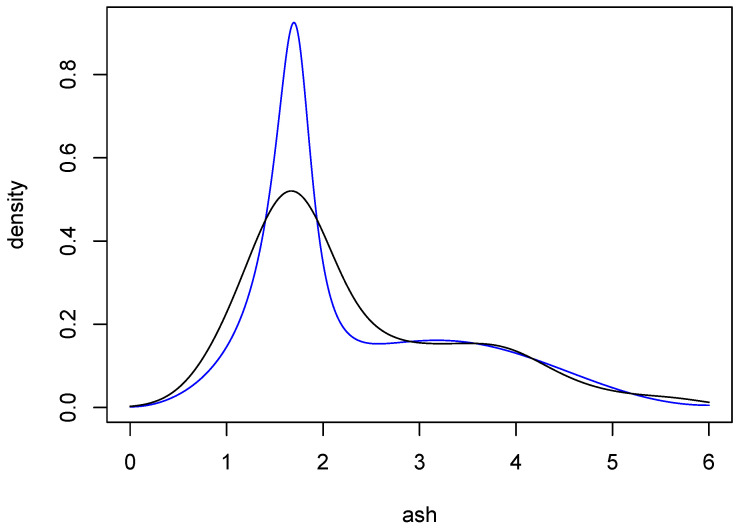
Density estimates for wine attribute data. The black curve is a Gaussian-kernel density estimate with normal reference bandwidth. The blue curve is an average of ARMA estimates resulting from the procedure described in Section 6.

Data on wine attributes are available from the UC Irvine Machine Learning Repository. The data “are the results of a chemical analysis of wines grown in the same region in Italy but derived from three different cultivars”, and have been analyzed in [14]. Here, we consider the attribute ash, which consists of 177 observations. The black curve in Figure 8 is a Gaussian-kernel density estimate for these data, with bandwidth chosen by the normal reference method [5] (p. 45).

Our Bayesian ARMA methodology was used to find a point estimate and confidence bands for the underlying density. The data lie on the interval (0,6), and were thus rescaled to the interval (0,π) before being analyzed. One hundred thousand draws from the posterior of each of the models ARMA(2,q), q=0,1,…,10, were obtained. The same algorithm and proposal distributions as in our simulation were used, with s=0.06 in each case. Both BIC and the principled Bayesian approach were used to identify good choices for *q*. For the latter, marginal likelihoods were calculated via importance sampling based on multivariate normal proposal distributions. For a given *q*, the mean and covariance matrix of the multivariate normal were the sample mean and covariance of the MCMC output for that *q*. Excellent approximations of marginal likelihoods were obtained by sampling at least one million times from each multivariate normal proposal distribution.

Two sets of approximate posterior probabilities are given in Table 4. One was obtained using BIC and the other with importance sampling, where the prior model probabilities were all 1/11. The two distributions are in remarkable agreement, both being maximized at q=4. The evidence for that choice of *q* is quite strong, with the probability being more than 0.80. Nonetheless, to obtain a point estimate of the underlying density, we used model averaging as follows: At each *q*, an ARMA density estimate was obtained by averaging over the 100,000 parameter sets generated by MCMC. A weighted average of these 11 estimates was then computed, with the weights being the exact posterior probabilities in Table 4. The result is the blue curve in Figure 8. This estimate is comparable to the kernel estimate, but interestingly the main peak of the ARMA estimate is more sharp than the corresponding peak of the kernel estimate. It is well known that kernel estimates tend to undershoot sharp peaks, and so the ARMA estimate seems to provide a compelling alternative.

One of the most attractive aspects of Bayesian methodology is its built-in means of assessing uncertainty. If we assume that the true density can be well approximated by an ARMA(2,10) density, then our procedure can be used to find confidence bands for said density. To obtain these bands, we began by repeating the following process, independently, one million times: A value of *q* is randomly chosen according to the exact distribution in Table 4, and then a set of parameters, i.e., an ARMA density, is selected from the MCMC output for that *q*. Using the densities so obtained, it is straightforward to approximate *pointwise* posterior probability intervals at a fine grid of *x*-values for any given probability. Call the two curves resulting from this process ℓ(x) and u(x), the lower and upper sets of intervals, respectively. One may now determine the proportion, *p*, of the 1,000,000 selected densities that lie completely between ℓ(x) and u(x), in which case *ℓ* and *u* are posterior probability *bands* of level *p*.

**Figure 9:**
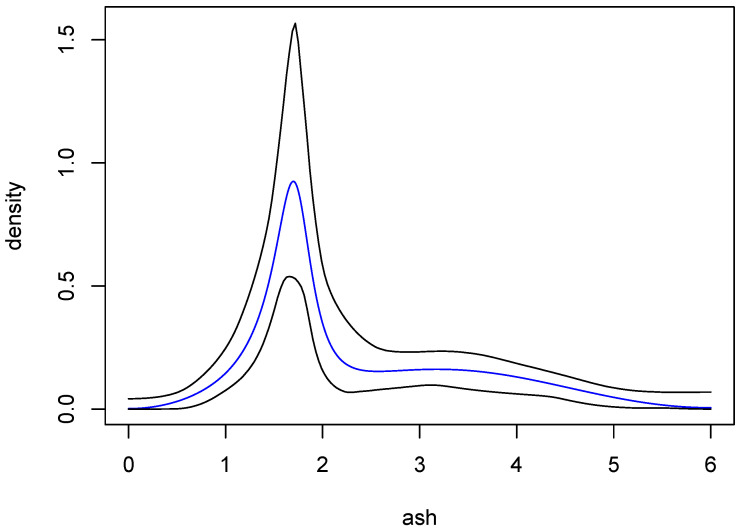
Approximate 95% posterior probability bands for the density of the ash variable. The black curves are the bands and the blue curve is the same ARMA estimate as in Figure 8.

The procedure just described was carried out with a grid of 200 evenly spaced points on the interval (0,π). Using various choices of probability levels for the pointwise intervals, it was found that when the pointwise probability level was 0.998, the corresponding *ℓ* and *u* contained more than 95% of the 1,000,000 ARMA estimates. The (approximate) 95% posterior probability bands so obtained are shown in Figure 9, along with the ARMA estimate from Figure 8. In fairness, the kernel estimate from Figure 8 also lies within these bands.

## 7. Concluding Remarks

We have proposed a Bayesian implementation of ARMA probability density estimators. In general, ARMA estimators are motivated by their parsimony and potential super-efficiency [1] relative to Fourier series estimators. A simulation study provided strong evidence that our ARMA estimators do indeed attain super-efficiency. An advantage of the Bayesian approach is that it allows straightforward assessments of uncertainty using the output from MCMC. In particular, posterior probability bands for the underlying density are possible, as was illustrated using wine attribute data. The focus in this paper was on ARMA estimators with AR order equal to 2, but higher order AR representations may also be useful.

## Figures and Tables

**Table 1:** Integrated squared error of various approximants. A cosine series with *q* terms is denoted $f_{q}$. The parameters of the MA and ARMA approximations were chosen to minimize ISE.

Approximant	ISE
$f_{8}$	0.10230
$f_{12}$	0.01027
MA(8)	0.24890
MA(12)	0.03493
ARMA(4,2)	0.02888
ARMA(6,2)	0.01167

**Table 2:** Distribution of data-driven moving average orders in the simulation. Each table value is the number of times an order was chosen out of 20 replications.

	Moving Average Order						
	4	5	6	7	8	9	10
**BIC**	18	0	1	0	1	0	0
**Laplace **	0	3	2	1	12	2	0

**Table 3:** Integrated squared error summary of the simulation with wrapped exponential density. The moving average order ${\tilde{m}}_{n}$ is the asymptotically optimal choice [1]. Simple FS refers to the truncated Fourier series, which used an asymptotic approximation to the MISE optimal truncation point. The numbers in the column “Optimum FS” are asymptotic approximations to the smallest possible mean integrated squared error for a tapered Fourier series.

	ARMA$\left(1,{\tilde{m}}_{n}\right)$		ARMA$\left(2,{\tilde{m}}_{n}\right)$		Simple FS		Optimum FS
n	Mean	Median	Mean	Median	Mean	Median	MISE
250	0.01147	0.00816	0.01262	0.00840	0.01366	0.01161	0.01338
500	0.00614	0.00480	0.00691	0.00515	0.00838	0.00709	0.00795
1000	0.00374	0.00284	0.00390	0.00295	0.00505	0.00437	0.00473
2000	0.00218	0.00172	0.00225	0.00176	0.00311	0.00278	0.00281

**Table 4:** Posterior probabilities of model orders for wine attribute data. Probabilities in the first row were approximated using BIC, while those in the second row are essentially exact, being approximated with use of importance sampling. The probabilities for 0 and 10 were 0 to three decimal places.

*q*	1	2	3	4	5	6	7	8	9
**BIC**	0.043	0.008	0.002	0.829	0.109	0.010	0.001	0.000	0.000
**Exact**	0.008	0.002	0.001	0.817	0.126	0.034	0.008	0.003	0.001

## Data Availability

The original contributions presented in this study are included in the article. Further inquiries can be directed to the corresponding author.

## References

[1] Hart J.D. (1988). An ARMA type probability density estimator. Ann. Stat..

[2] Hart J.D., Gray H.L. (1985). The ARMA method of approximating probability density functions. J. Statist. Plann. Inference.

[3] Carmichael J.P. (1984). Consistency of an autoregressive density estimator. Math. Operationsforsch. Stat. Ser. Stat..

[4] Aydin D., Yilmaz E., Chamidah N. (2021). Rational (Padé) approximation for estimating the components of the partially-linear regression model. Inverse Probl. Sci. Eng..

[5] Silverman B.W. (1986). Density Estimation for Statistics and Data Analysis.

[6] Woodward W.A., Gray H.L., Elliott A.C. (2012). Applied Time Series Analysis.

[7] Watson G.S., Leadbetter M.R. (1963). On the estimation of the probability density. I. Ann. Math. Stat..

[8] Watson G.S. (1969). Density estimation by orthogonal series. Ann. Math. Stat..

[9] Jammalamadaka S.R., Kozubowski T.J. (2004). New families of wrapped distributions for modeling skew circular data. Commun. Stat.—Theory Methods.

[10] Barndorff-Nielsen O., Schou G. (1973). On the parametrization of autoregressive models by partial autocorrelations. J. Multivar. Anal..

[11] Barnett G., Kohn R., Sheather S. (1996). Bayesian estimation of an autoregressive model using Markov chain Monte Carlo. J. Econom..

[12] Small C.G. (2010). Expansions and Asymptoics for Statistics.

[13] R Core Team (2025). R: A Language and Environment for Statistical Computing.

[14] Aeberhard S., Coomans D., de Vel O. (1994). Comparative analysis of statistical pattern recognition methods in high dimensional settings. Pattern Recognit..

